# Stage IV Colorectal Cancer Patients with High Risk Mutation Profiles Survived 16 Months Longer with Individualized Therapies

**DOI:** 10.3390/cancers12020393

**Published:** 2020-02-08

**Authors:** Alexander Hendricks, Anu Amallraja, Tobias Meißner, Peter Forster, Philip Rosenstiel, Greta Burmeister, Clemens Schafmayer, Andre Franke, Sebastian Hinz, Michael Forster, Casey B. Williams

**Affiliations:** 1 Department of General and Thoracic Surgery, University Hospital Schleswig-Holstein Campus Kiel, 24105 Kiel, Germany; alexander.hendricks@med.uni-rostock.de (A.H.); greta.burmeister@med.uni-rostock.de (G.B.); clemens.schafmayer@med.uni-rostock.de (C.S.); sebastian.hinz@med.uni-rostock.de (S.H.); 2 Department of Molecular and Experimental Medicine, Avera Cancer Institute, Sioux Falls, SD 57105, USA; anu.amallraja@avera.org (A.A.); tobias.meissner@avera.org (T.M.); 3 McDonald Institute for Archaeological Research, University of Cambridge, Cambridge CB2 1TN, UK; pf223@cam.ac.uk; 4 Institute of Clinical Molecular Biology, Christian-Albrechts-University of Kiel, 24105 Kiel, Germany; p.rosenstiel@mucosa.de (P.R.); a.franke@mucosa.de (A.F.)

**Keywords:** metastatic colorectal cancer, mutational landscape, treatment, overall survival

## Abstract

Personalized treatment vs. standard of care is much debated, especially in clinical practice. Here we investigated whether overall survival differences in metastatic colorectal cancer patients are explained by tumor mutation profiles or by treatment differences in real clinical practice. Our retrospective study of metastatic colorectal cancer patients of confirmed European ancestry comprised 54 Americans and 54 gender-matched Germans. The Americans received standard of care, and on treatment failure, 35 patients received individualized treatments. The German patients received standard of care only. Tumor mutations, tumor mutation burden and microsatellite status were identified by using the FoundationOne assay or the IDT Pan-Cancer assay. High-risk patients were identified according to the mutational classification by Schell and colleagues. *Results*: Kaplan–Meier estimates show the high-risk patients to survive 16 months longer under individualized treatments than those under only standard of care, in the median (*p* < 0.001). Tumor mutation profiles stratify patients by risk groups but not by country. *Conclusions*: High-risk patients appear to survive significantly longer (*p* < 0.001) if they receive individualized treatments after the exhaustion of standard of care treatments. Secondly, the tumor mutation landscape in Americans and Germans is congruent and thus warrants the transatlantic exchange of successful treatment protocols and the harmonization of guidelines.

## 1. Introduction

Despite extensive efforts in colorectal cancer screening [1], colorectal cancer is a growing major health burden that will, according to estimates, account for 1.1 million cancer deaths annually by 2030 [2]. Even though considerable progress in the treatment of the disease has been achieved, the average five-year survival rate is below 70%. Nearly 25% of patients have distant metastases at the time of the diagnosis. In this latter group of patients, the five-year overall survival rate is below 20% [3]. Given this dismal outlook, a key question for the clinician and the patient is whether to follow standard-of-care guidelines or embark on a molecularly guided approach. Therefore, our study investigates the hypothesis that different treatment strategies can explain the different overall survival lengths of individual patients. To rule out sampling bias, we selected clinically well-characterized and sex-matched patients of European descent, from both sides of the Atlantic. To rule out mutational bias, we investigated whether there were significant differences in the mutational landscapes of the American vs. German colorectal cancers, and we then classified subgroups of metastatic patients with colorectal cancer, according to the mutational classification proposed by Schell and colleagues [4]. The Schell classification for a patient’s colorectal cancer is based on the combination and number of mutations in the colorectal cancer driver genes *APC*, *TP53*, and *KRAS*. While *TP53*, *KRAS,* and *NRAS* are routinely tested for mutations, *APC* is not yet generally tested, despite the high frequency of mutant *APC*. About 80% of sporadic colorectal cancers harbor truncating mutations in *APC* (frameshift, nonsense, and splice site mutations), which lead to polyposis originating from single epithelial stem cells in the colonic crypt that ultimately progresses to cancer [5,6]. Truncating mutations in *APC*, combined with loss of *TP53*, leads to chromosomal instability with extensive aneuploidy [6]. RAS mutations are commonly found in hyperproliferating cells [5]. The highest risk for poor outcome and survival was found in Schell Class 4 cancers (two or more truncating *APC* mutations plus mutations in *TP53* and *KRAS*), followed by Schell Class 0 (no truncating *APC* mutations) [4].

Our main aims were to compare the overall survival for individualized treatment versus only standard-of-care treatment, including the overall survival for American treatment versus German treatment in our real clinical practice. The American patients at the Avera Cancer Institute received standard-of-care (SOC) treatment until failure. For 35 of the American patients, the SOC treatment was followed by individualized treatment with extensive molecular testing and case discussions in a molecular tumor board. The German patients at the University Hospital Schleswig-Holstein were strictly treated according to the German SOC guidelines that were in effect at the time of the individual patient’s diagnosis and treatment. Although the guidelines are subject to frequent updates, the American SOC usually comprises a larger choice of clinical tests and treatments than the German SOC at any given date. Even more so, the American SOC that we followed in 2008–2019 is more individualized than the German SOC that we followed half a decade earlier, and thus American vs. German overall survival times allowed us to validate whether individualized precision medicine may be preferential to SOC, without the ethical dilemma of withholding the best available treatments to a patient.

Our study results suggest that there were no significant differences in ethnicity, ancestry, gender composition or mutational landscapes between the American and German patients. This leaves the SOC differences as a plausible explanation for the significantly extended survival of the American patients. After stratification by mutational classification according to Schell and colleagues [4], low-risk patients did not seem to benefit from individualized precision medicine, but high-risk patients benefited significantly.

## 2. Results and Discussion

**Figure 1:**
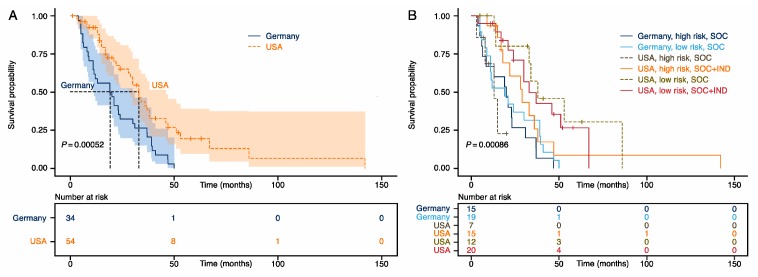
Kaplan–Meier estimates for stage IV colorectal cancer patients according to country and treatment regimens. (**A**) Patients in America diagnosed in 2008–2017 had a median survival probability of 33 months, compared to 19.5 months in patients in Germany diagnosed in 2003–2010. Shading indicates 95% confidence intervals. (**B**) Kaplan–Meier curves stratified by country, standard of care (SOC), and SOC, followed by individualized treatments (SOC + IND), and mutational high risk (Schell classes 0 and 4) vs. low risk (Schell classes 1–3). NB: The American SOC between 2008 and 2017 was more individualized than the German SOC between 2003 and 2010.

**Figure 2:**
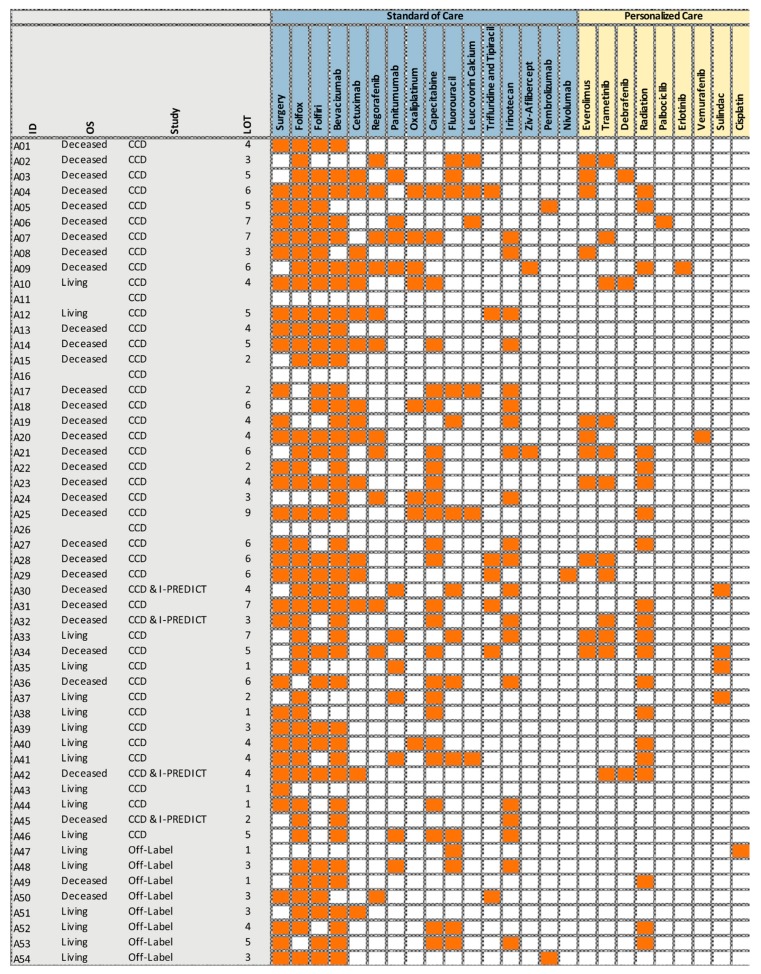
Treatments received by the American patients (A01–A54) in 2008–2017.

**Figure 3:**
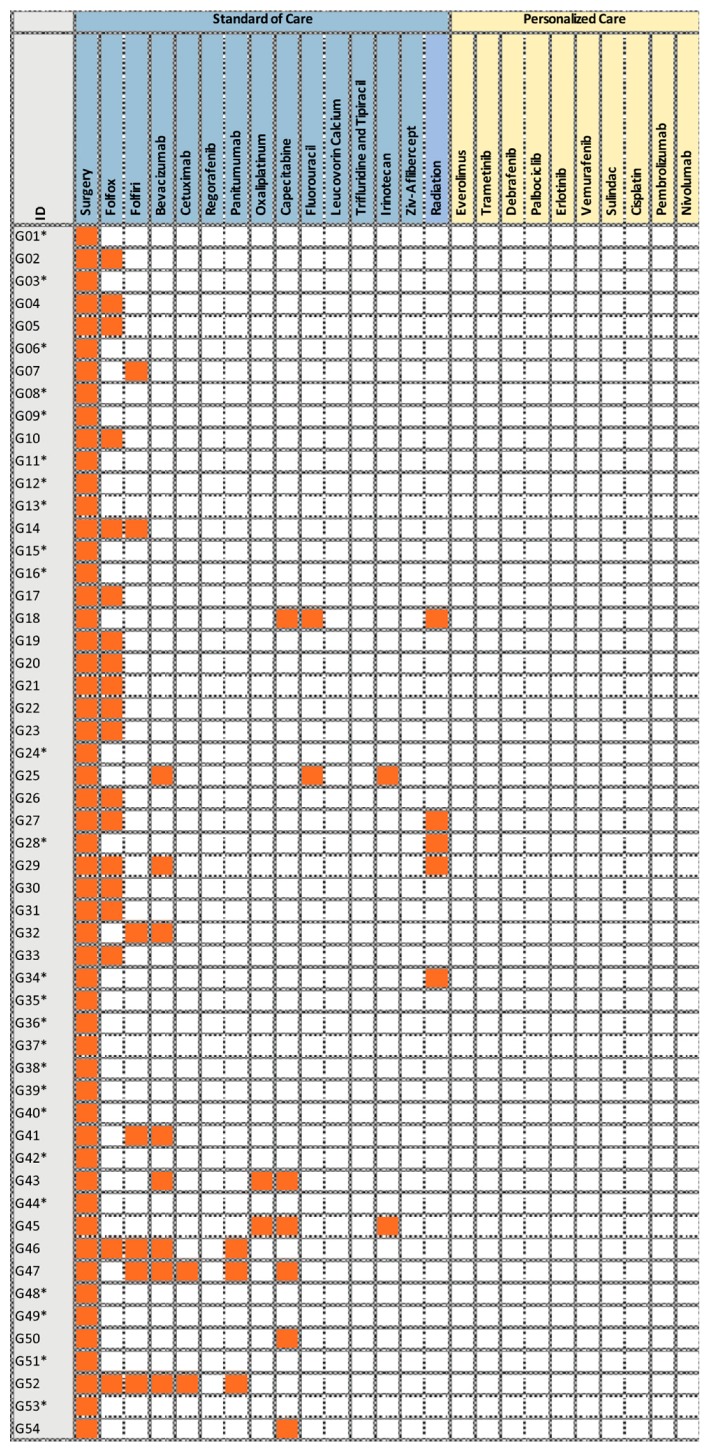
Treatments received by the German patients (G01–G54) in 2003–2010. The asterisk (*) marks patients who received chemotherapy at external oncological practices.

**Figure 4:**
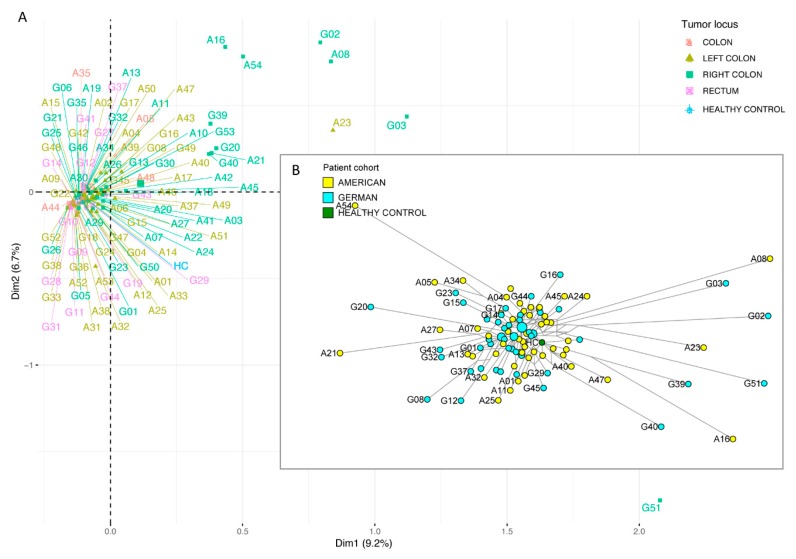
Mutational landscape analysis of metastatic colorectal cancer patients, based on mutated genes. (**A**) Principal component analysis and (**B**) phylogenetic network analysis independently show that microsatellite stable cancers group together while the highly mutated microsatellite instable cancers are distinct outliers, with individually mutated genes. Importantly, there is no mutational separation into American vs. German patient groups.

**Figure 5:**
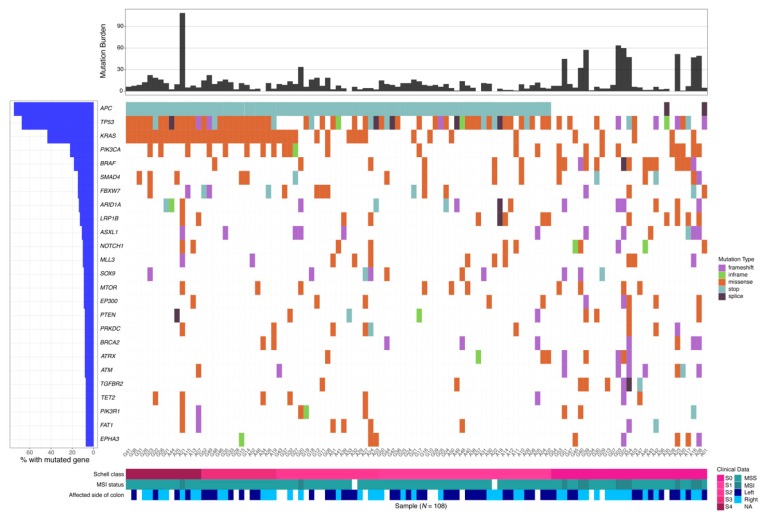
Mutational landscape of metastatic colorectal cancer patients. Each patient is represented by a column, showing mutated genes, tumor mutation burden, Schell class, microsatellite instability status, and side of the colorectal cancer.

To answer the key question whether individualized medicine is preferential over standard-of-care treatments for stage IV colorectal cancer patients, we compared the overall survival times (Figure 1) for individualized treatments versus SOC, and for American SOC versus (less individualized) German SOC (Figure 2 and Figure 3). We show that the mutational landscapes in colorectal cancer tissue are congruent in Americans and Germans (Figure 4 and Figure 5). We also show that our entire cohort is of Northern European ancestry (see subsection entitled Demographics). This leaves treatment differences as a plausible explanation for the observed survival differences. Specifically, our survival analysis indicates that the Americans are benefiting from their SOC regimens, and that American patients with high-risk mutational profiles are benefiting from individualized treatments.

### 2.1. Demographics

We analyzed a total of 108 patients diagnosed with histologically confirmed colorectal cancer. All patients showed late-stage disease (stage III, *N* = 20 or stage IV, *N* = 88) at the time of diagnosis and analysis. Ninety-three patients were diagnosed with colon cancer, and 15 patients with rectal cancer. To minimize potential gender selection bias, the American and German cohorts were sampled for best gender matching. The median age of the combined cohorts was 64 years (range: 25–95 years). An overview and synopsis of the descriptive and clinical data are given in Table 1 and Table S1.

Demographic analysis suggested that the American cohort is European by descent, and thus of comparable ethnicity and hereditary genomic composition to the German cohort. In detail, for the Avera patients, surname information was available for three-quarters of the cohort. A comparison with the global Y-chromosome and surname database maintained by Roots for Real (Cambridge, United Kingdom) indicated that the American cohort represented a population sample of European descent, with only two potential exceptions (one Jewish surname and one Mexican/Spanish surname). There was a predominance of Northern European surnames, with 30% deriving from the British Isles, 24% from the German-speaking and Benelux countries, 6% from Scandinavia, and 6% from Eastern Europe. The remaining surnames were of general European descent.

The German patients were recruited in the North German region of Schleswig-Holstein, which historically consisted of Danish-, German-, and Slavonic-speaking populations. In addition, there have been minor migration events in recent centuries [7], but the major event was the settlement of World War II refugees from Eastern German territories of what is now Poland, nearly doubling the population of Schleswig-Holstein after 1945. Genealogical surname analysis indicates that the German cohort is of two-thirds German descent, one-fifth Slavic (East German and Polish) descent, and one-tenth Danish descent. This means that there is no fundamental difference from the American cohort.

### 2.2. Precision Medicine Increased Overall Survival in High-Risk Patients

Figure 1 shows Kaplan–Meier survival estimates for our patients. As expected, our patients treated in the USA show improved overall survival (OS) compared to our patients treated in Germany almost a decade earlier (Figure 1A). The American patients show an improved OS by a median of 13.5 months (Germany = 19.5 months; USA = 33 months; *p* < 0.001; hazard ratio (HR) = 0.43; 95% confidence interval (CI) 0.26–0.70). Subsequent stratification into SOC vs. individualized care (IND) after SOC shows that the survival benefit is predominantly due to differences in the SOC between USA and Germany almost a decade earlier (median OS: Germany SOC = 19.5 months; USA SOC = 34, USA IND: 33; Figure 1B). To investigate whether specific subgroups of patients benefit from individualized treatment after exhausting SOC options, we further stratified patients into low and high risk. We defined ‘high risk’ as those patients classified by Schell et al. [4] into either group 0 (*APC* wild type) or group 4 (*APC* with two or more truncating mutations, *TP53* mutated, *KRAS* mutated). We defined ‘low risk’ as classified into groups 1–3. This analysis revealed that American high-risk patients gained a median 16-month survival benefit when treated with individualized approaches, compared to high-risk patients that received American SOC (29 vs. 13 months) (Figure 1B). Low-risk patients did not seem to have benefited from individualized approaches after SOC (Figure 1B). In a multivariate analysis, including the covariates age, gender, tumor location, microsatellite status, treatment, and risk stratification, receiving treatment in the US in 2008–2019 remained an independent prognostic factor (*p* < 0.01, HR: 0.29, CI 0.11–0.75). In addition, being male was associated with prolonged OS (*p* = 0.005, HR = 0.38, CI: 0.20–0.75), and right-sided tumors were associated with worse outcome (*p* = 0.04, HR = 1.88, CI: 1.02–3.49). Age was not associated with OS. This is congruent with Virostko and colleagues’ recent finding that there is little age-related difference in survival for patients who survive longer than 90 days after surgery [8].

#### Individualized Treatment Implementation

The American patients were either enrolled into the multi-center clinical trials ‘Identifying Molecular Drivers of Cancer (CCD)’ (NCT02470715), I-PREDICT (NCT02534675) [9], or treated off-label with molecularly guided therapies. Our hypothesis is that matching a single agent to a heterogeneous tumor with multiple genomic alterations will not succeed in improving treatment outcomes, and hence combinations of customized agents are needed for a majority of patients with advanced solid tumors. Targeted therapies were given either after exhaustion or in addition of SOC options and included individualized combinations of one or more conventional cytotoxic compounds with one or more targeted drugs (immunotherapies, antibodies and/or small molecule inhibitors, Table S2 and Figure 2).

Therapies were selected by incorporating recommendations of a molecular tumor board consisting of oncologists, pharmacists, nurses, genetic counselors, bioinformaticians, patient advocates, and molecular biologists. The therapies ultimately given to patients were furthermore based on the treating physician’s consideration of patient preferences, drug toxicities, and availability (i.e., insurance coverage). For administration of drug combinations, we routinely followed Nikanjam et al. [10], or other data where available. Patients generally did not receive treatment without at least safety data being available. In addition, patients were monitored closely, and adverse event management was planned on the basis of theoretical drug metabolism, with the result that no treatment-related mortality occurred.

For comparison, the German SOC received by our German patients are given in Table S3 and Figure 3.

### 2.3. Congruent Cancer Mutation Landscape in Americans and Germans

To test for potential differences in the mutational landscapes of American vs. German patients, three different methods were used: (i) a mutational classification according to Schell et al. [4] based on *APC*, *TP53*, and *KRAS* mutations; (ii) principal component analysis; and (iii) phylogenetic network analysis.

The American versus German stage IV cohorts show no significant difference according to the Schell classification (Table 1) with *p* = 0.69 (Fisher’s exact test). Based on the Schell classification, neither our American cohort nor our German cohort are significantly different to the published Schell cohort of Americans, with *p* = 1.00 and *p* = 0.48, respectively. Our German cohort versus the combined cohort of our American patients plus the Schell cohort show no significant difference, with *p* = 1.00.

The principal component analysis (PCA) and the phylogenetic network analysis (Figure 4) are based on somatically mutated genes within the shared set of genes used for both cohorts. For this analysis, we also included stage III patients (*N* = 20). Both analyses show that Americans and Germans are intermingled with each other, i.e., not different from each other. Our 54 American patients were tested by using the FoundationOne panel (Table S4), of which 52 patients had mutations in the shared set of genes. Our 54 German patients were tested, using the IDT Pan-Cancer panel (Table S4), of which 51 patients had mutations in the shared set of genes. In Figure 4A, the PCA shows a microsatellite stable (MSS) cluster and two microsatellite instable (MSI) clusters. There is no separation into American vs. German clusters. In Figure 4B, the network analysis shows a phylogeny, with its root in a healthy node near the network center, a cluster of patients with low mutation load near the center, and “rings” of patients with higher mutation loads more distant from the root. The network analysis shows no separation into American vs. German branches. Patients with MSI and a high number of private mutations are shown as nodes at each end of their individual, long phylogenetic branches (Figure 4B and Figure S1). The network shows a distinct sector of RAS-mutated patients *(KRAS* and/or *NRAS)* (Figure S1), a cluster of 8 *BRAF*-mutated MSS patients (8%), and 8/11 MSI patients with mutated *BRAF* (73%). The most recent German S3 guideline on colorectal cancer (v2.1, 2019) recommends a first-line treatment of FOLFOXIRI for *BRAF*-mutated patients, or their inclusion into a clinical trial with innovative treatments.

Figure 5 summarizes the landscape of mutations, TMB, MSI status, Schell classification, and tumor location for each patient’s tumor sample. It shows that MSI is strongly associated with Schell class 0, right-sided tumors, and lack of *APC* mutations, confirming previous reports [4]. Table S5 and Table S6 summarize the somatic mutations detected in the Americans and Germans, in the shared set of genes (Table S4). Sequencing coverage statistics for the FoundationOne and IDT panels are given in Table S7.

Two recent studies on colorectal cancer patients—one comparing Japanese and American cohorts [11], and another comparing Brazilian patients to multiple international cohorts [12]—found that even if mutations in certain driver genes were enriched in specific ethnic subpopulations, the overall mutational landscape of colorectal cancer is comparable. This is clearly supported by multiple methods in our study with American and German patients.

### 2.4. NGS-Based MSI Test Congruent with Clinical MSI Test

To assess the validity of NGS-based MSI testing on our American and German patient cohorts, the NGS results were compared with clinical MSI results, where available. Our NGS and clinical MSI test results (Table S1) show that NGS correctly classified our cohorts’ samples into MSI or MSS. However, without our TMB-based correction, the NGS-based MSIsensor tool incorrectly classified one of the samples as MSI-L instead of MSS. From the clinical side, 10 of the 11 MSI tumors originated in the right colon (91%). MSI colon cancers tend to evolve from large but flat precursors—sessile serrated adenomas (SSAs)—which are more difficult to detect than polyps [13,14]. Resections of SSAs are difficult, with incomplete resections reported for up to 48% of cases [14]. Due to the large diameter of the proximal colon, such patients may be asymptomatic until the tumor has metastasized, highlighting the dangers of MSI colorectal cancers and their precursor lesions. While MSI is a biomarker indicating immune checkpoint inhibition in the USA [15], the German S3 guideline recommends that the first line of treatment should be based on RAS mutation status. Of note, MSI colorectal cancers usually have no *APC* mutations and thus fall into the ‘high-risk’ Schell group 0, for which we have shown above that individualized treatments appear to have a significant survival benefit over SOC.

## 3. Patients

The study was approved by Avera IRB (#2019.005/100572) and by the University of Kiel medical faculty ethics board (#A110/99).

Table 1 summarizes our patients. Patients were included who survived 90 days or longer after surgery. The American cohort comprised 54 patients with metastatic colorectal cancer at the Avera Cancer Institute. They presented to the Institute between 2008 and 2017 with heavily pretreated stage IV CRC. All American patients included in this study were well enough to receive further treatment. At the Avera Cancer Institute, they then received American SOC, and after failure, individualized drug combinations, depending on insurance coverage. The German cohort comprised 54 patients with metastatic colorectal cancer from the Biomaterialbank des Krebszentrums Nord (BMB-CCC). Their patient-matched tumor/normal fresh-frozen tissue sample pairs were sequenced and analyzed by the authors as described below. The German patients were diagnosed between 2003 and 2010. All German patients received SOC therapies according to the German guidelines. The stage IV patients were used for the survival analysis, and the stage III and stage IV patients were used for the mutational landscape analyses, as detailed further in the Methods. No American stage III colorectal cancer patients were available for the study.

## 4. Materials and Methods

### 4.1. Foundation Medicine Routine Clinical Testing of the American Patients

Targeted DNA sequencing was performed using the FoundationOne assay (Cambridge, MA, USA), as described in [16]. Briefly, sequencing libraries were prepared from >50 ng DNA extracted from FFPE samples with a minimum of 20% tumor content. Hybridization capture was performed, and the libraries underwent paired-end 49 bp sequencing to a median coverage of >500X on the Illumina platform. The Foundation Medicine sequencing data have not been archived in a public human sequence archive because the patients did not consent.

### 4.2. Retrospective Next-Generation Sequencing of the German Patients

DNA was isolated from fresh-frozen tissue, using AllPrep DNA-RNA-miRNA Universal Kit (QIAGEN #80224). 108 Illumina TruSeq Nano libraries were prepared from 100 ng DNA each according to protocol. Hybridization capture was performed using the IDT xGen Pan-Cancer panel v1.5, which targets cancer genes identified by The Cancer Genome Atlas (TCGA). Sequencing was performed on Illumina NextSeq, using 2 × 150 bp paired-end reads. We have securely archived the fastq sequencing files at the European Genome-Phenome Archive (EGA) under study accession ID EGAS00001004108. The EGA is subject to the EU’s General Data Protection Regulation and access to the data may be applied for, subject to a data-access agreement with project description and ethics board approval.

### 4.3. Bioinformatic Analysis of the German Patients

Raw sequencing data were aligned to the genome (hs37d5) with BWA-MEM (v 0.7.15) (https://arxiv.org/abs/1303.3997) and realigned using ABRA (v 0.97) [17]. Duplicates were marked using sambamba (v 0.6.3) [18]. Somatic variants were called, using VarDict (v. 1.5.1) [19], and annotated by using ANNOVAR (v Feb 2016) [20].

Technical filters were applied to the mutation calls: minimal variant depth of 7, minimal base quality of 30, minimal variant allele frequency of 0.003, strand bias according to the VarDict test. Variants were required to have a minimal depth of 10 in either the tumor or matched normal sample. Variants caused by DNA damage were filtered out, as recommended in [21] and [22]. Additional filtering was done on low-frequency variants that have low depth, as shown by (http://bcb.io/2016/04/04/vardict-filtering/). ExAC and 1000 genomes databases were used to filter variants with a population allele frequency threshold of 0.01% [23,24].

Tumor mutational burden (TMB) was calculated as the number of mutations per 1 Mb relative to the panel size and rounded to the first decimal place.

Microsatellite instability (MSI) was assessed by using MSIsensor (v 0.5) on matched tumor-normal samples. The cutoffs used were as follows: score <10 for MSS (microsatellite stable), score between and including 10 and 30 for MSI-L (low), and score >30 for MSI-H (high). When the scores generated by MSIsensor were close to a cutoff value, the corresponding TMB value of the sample was utilized to make a final judgement on the classification (Supplementary Table S1). If the TMB value was high, it was classified as MSI, as supported by the evidence shown in a previous study [25].

Sample-pairing validation was performed, as previously published, by comparing polymorphism signatures between all samples [26]. Somatic single nucleotide substitutions were validated by using pibase [26]. Somatic indels were manually validated by using IGV [27].

### 4.4. Comparison of Mutational Signatures in Americans vs. Germans

According to Strickler and colleagues, a threshold of 25% of the maximal tumor allele frequency in a tumor sample was applied to classify a mutation as clonal or subclonal [28]. Subclonal mutations were not counted if they had less than 1/10 of the maximal tumor allele frequency in the sample, or less than 3% absolute tumor allele frequency.

To answer the question whether the American and German cohorts had congruent mutational signatures or not, the cohorts’ mutations were compared at three levels of resolution.

For the first level of resolution, we used the mutational classification proposed by Schell and colleagues [4]: 0—no truncating mutations in *APC*; 1—one truncating *APC* mutation, and *TP53* or *KRAS* mutated but not both; 2—two truncating *APC* mutations, and *TP53* or *KRAS* mutated but not both; 3—one truncating *APC* mutation, and both *TP53* and *KRAS* mutated; 4—two truncating *APC* mutations, and both *TP53* and *KRAS* mutated.

For the second level of resolution, we performed a principal component analysis, and for the third level of resolution, we performed a phylogenetic network analysis [29]. We analyzed the same data in the second and third levels of resolution. We considered the genes contained in the overlap of the FoundationOne panel and the IDT panel (Table S4). We considered only the clonal mutations. A mutated gene in a patient was scored as 1, and a wild-type gene scored as 0. If there were patients with more than one mutation in a gene, then the affected gene names were duplicated so that the binary 1/0 scoring system could be used, e.g., *APC*, *APC_1*, *APC_2*, *TP53*, and *TP53_1.*

### 4.5. Microsatellite Instability Testing

MSI tests for all German tumor and normal samples were performed by the Department of Pathology in Kiel, using five mononucleotide markers (BAT25, BAT26, NR21, NR24, and MONO27).

MSI testing for the American patients was carried out as part of the FoundationOne panel, where available. For the samples that did not have those results, MSIsensor (v 0.5) was used to perform MSI calling on tumor samples only, as we did not have matched normals for this cohort. This method was able to resolve the samples as either MSS or MSI, based on a cutoff score of 25, but not the grade of MSI, as shown previously [30]. Additionally, this was validated in over a thousand samples from Avera with FoundationOne tests. As described above, when scores were close to the cutoff value, MSI classification was adjusted.

## 5. Conclusions

High-risk patients may be identified as having Schell mutational classifications 0 and 4. These patients may survive significantly longer if they receive individualized treatments after the exhaustion of standard-of-care treatments. Secondly, our study has, for the first time, proven what has previously often just been assumed: The mutational landscapes in American and German metastatic colorectal cancer patients are comparable—on the basis of Schell profiles, principal components, and phylogeny—despite the geographic and environmental divergence. However, we find that the overall survival in American patients who received standard-of-care treatments or individualized targeted treatments once they failed standard therapies is significantly longer than that of German patients who received less individualized SOC almost a decade earlier. Therefore, we also suggest that innovative treatments should and can be readily harmonized and exchanged between American and German cancer centers.

## Figures and Tables

**Table 1:** Summary of clinical metadata and mutational classification, according to Schell et al.

Clinical Data	Total *N* (%)	American *N* (%)	German *N* (%)	*p*
Gender				
Male	61 (56.5)	31 (57.4)	30 (55.6)	
Female	47 (43.5)	23 (42.6)	24 (44.4)	1.00
Age [years]				
Median (Range)	64 (25–95)	60 (25–82)	68 (42–95)	
<55	26 (24.1)	18 (33.3)	8 (14.8)	
55–75	65 (60.2)	33 (61.1)	32 (59.3)	
>75	17 (15.7)	3 (5.6)	14 (25.9)	<0.001
Tumor site				
Colon	93 (86.1)	54 (100)	39 (72.2)	
right	46 (49.5)	28 (51.9)	18 (46.2)	
left	42 (45.2)	21 (38.9)	21 (53.8)	0.39
NA ^1^	5 (5.4)	5 (9.3)	0 (0)	
Rectum	15 (13.9)	0 (0)	15 (27.8)	
UICC stage ^2^				
III	20 (18.5)	0 (0)	20 (37.0)	
IV	88 (81.5)	54 (100)	34 (63.0)	
Lymph node status				
pN positive	74 (68.5)	24 (44.4)	50 (92.6)	
pN negative	NA	NA	4 (7.4)	
MSI ^3^				
Stable	95 (88)	47 (87)	48 (88.9)	
High	11 (10.2)	5 (9.3)	6 (11.1)	
NA	2 (1.8)	2 (3.7)	0 (0)	1.00
Schell-Classification ^4,**5**^				
Class 0	23 (26.1)	15 (27.8)	8 (23.5)	
Class 1	24 (27.3)	17 (31.5)	7 (20.6)	
Class 2	16 (18.2)	9 (16.7)	7 (20.6)	
Class 3	11 (12.5)	6 (11.1)	5 (14.7)	
Class 4	14 (15.9)	7 (13.0)	7 (20.6)	0.69

^1^ NA: not available; ^2^ UICC: Union internationale contre le cancer; ^3^ MSI: microsatellite instability; ^4^ only for stage IV patients; ^5^ percentages do not always add up to 100.0, due to rounding.

## Supplementary Materials

The following are available online at https://www.mdpi.com/2072-6694/12/2/393/s1. Figure S1: Phylogenetic network analysis details, Table S1: Patient metadata in detail, Table S2: Treatment combinations for American patients at Avera Cancer Institute, initial presentation in 2008–2017, Table S3: Treatment combinations for German patients at University Hospital Schleswig-Holstein, initial presentation in 2003–2010, Table S4: List of genes in Foundation Medicine One panel and in IDT xGen Pan-Cancer panel, Table S5: Clonal somatic tumor mutations detected in American patients in consensus gene panel (genes present in FoundationOne and IDT Pan-Cancer), Table S6: Somatic tumor mutations detected in German patients in consensus gene panel (genes present in FoundationOne and IDT Pan-Cancer), Table S7: Sequencing Statistics.
