# Supplementary material for: Stage IV Colorectal Cancer Patients with High Risk Mutation Profiles Survived 16 Months Longer with Individualized Therapies
Source: Cancers (Basel). 2020 Feb 8;12(2):393. doi: 10.3390/cancers12020393 (PMC7072525; doi:10.3390/cancers12020393)
Supplement: Supplementary file 1 [file cancers-12-00393-s001.zip › cancers-698395-2.revision.supplementary_materials/Supp_Fig_S1.pdf]

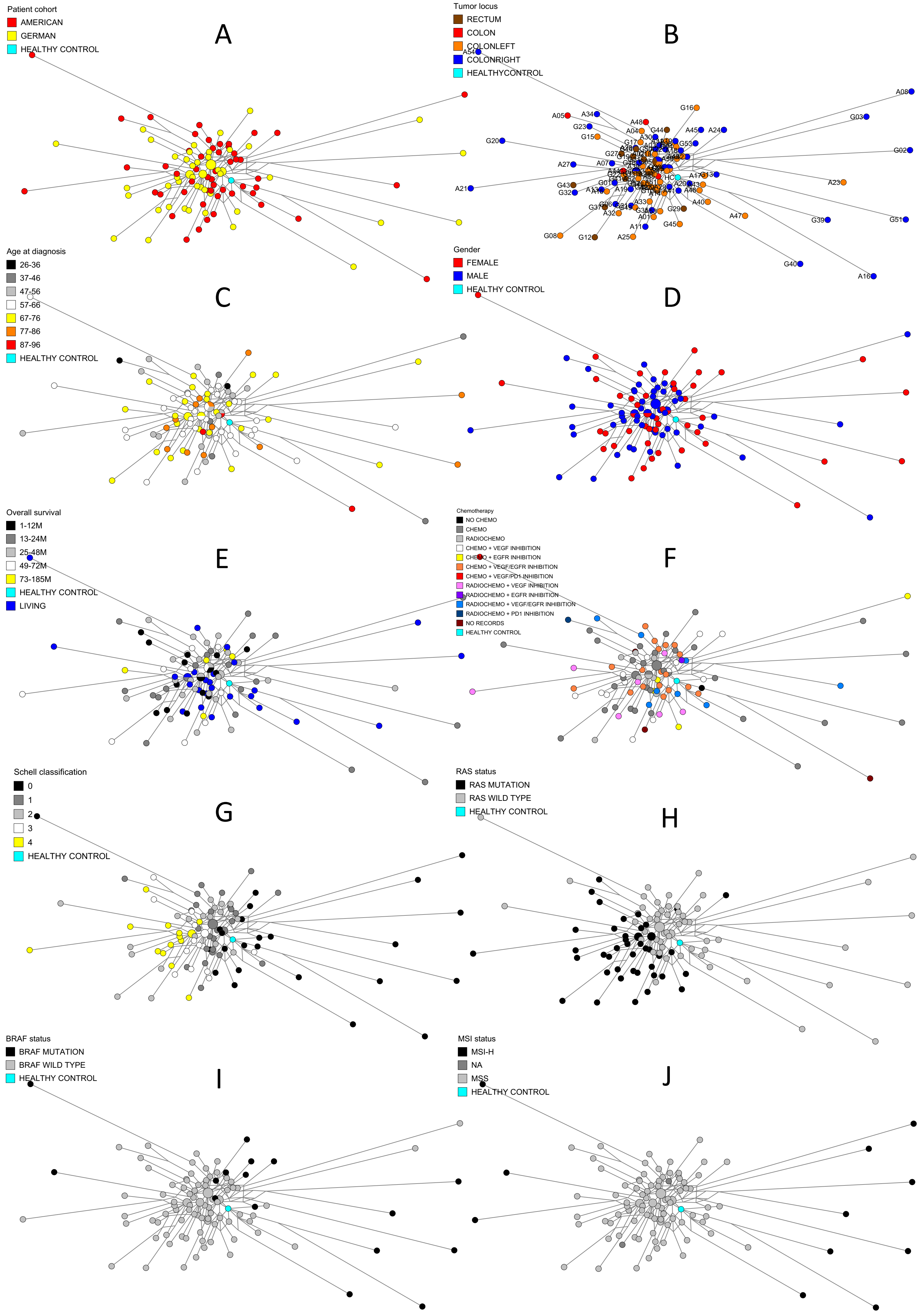

A
Patient cohort
AMERICAN
GERMAN
HEALTHY CONTROL
B
Tumor locus
RECTUM
COLON
COLONLEFT
COLONRIGHT
HEALTHYCONTROL
A54
A08
G16
A05
A34
G03
A48
G23
A04
G44
A45
A24
G15
G53
G20
G02
A27
A07
A23
G43
G32
A21
A17
G13
A20
A46
A40
A47
G39
G51
G37
A32
G29
G45
A11
G08
G12
A25
G40
A16
C
Age at diagnosis
26-36
37-46
47-56
57-66
67-76
77-86
87-96
HEALTHY CONTROL
D
Gender
FEMALE
MALE
HEALTHY CONTROL
E
Overall survival
1-12M
13-24M
25-48M
49-72M
73-185M
HEALTHY CONTROL
LIVING
F
Chemotherapy
NO CHEMO
CHEMO
RADIOCHEMO
CHEMO + VEGF INHIBITION
CHEMO + EGFR INHIBITION
CHEMO + VEGF/EGFR INHIBITION
CHEMO + VEGF/PD1 INHIBITION
RADIOCHEMO + VEGF INHIBITION
RADIOCHEMO + EGFR INHIBITION
RADIOCHEMO + VEGF/EGFR INHIBITION
RADIOCHEMO + PD1 INHIBITION
NO RECORDS
HEALTHY CONTROL
G
Schell classification
0
1
2
3
4
HEALTHY CONTROL
H
RAS status
RAS MUTATION
RAS WILD TYPE
HEALTHY CONTROL
I
BRAF status
BRAF MUTATION
BRAF WILD TYPE
HEALTHY CONTROL
J
MSI status
MSI-H
NA
MSS
HEALTHY CONTROL
